# Halved contrast medium dose coronary dual-layer CT-angiography – phantom study of tube current and patient characteristics

**DOI:** 10.1007/s10554-024-03062-6

**Published:** 2024-02-22

**Authors:** C. H. Kristiansen, P. M. Tetteroo, M. M. Dobrolinska, P. M. Lauritzen, B. K. Velthuis, M.J.W. Greuter, D. Suchá, P.A. de Jong, N.R. van der Werf

**Affiliations:** 1 Department of Diagnostic Imaging and Intervention, Akershus University Hospital, Lørenskog, Norway; 2 Department of Life Sciences and Health, Oslo Metropolitan University, Oslo, Norway; 3 Department of Radiology & Nuclear Medicine, University Medical Center Utrecht, Utrecht, The Netherlands; 4 Department of Radiology, University Medical Center Groningen, University of Groningen, Groningen, The Netherlands; 5 Division of Cardiology and Structural Heart Diseases, Medical University of Silesia in Katowice, Katowice, Poland; 6 Department of Radiology & Nuclear Medicine, Oslo University Hospital, Oslo, Norway; 7 Philips Healthcare, Best, The Netherlands

**Keywords:** Coronary angiography, Coronary artery disease, Computed tomography angiography, Contrast Media / administration & dosage, Phantoms, imaging

## Abstract

Virtual mono-energetic images (VMI) using dual-layer computed tomography (DLCT) enable substantial contrast medium (CM) reductions. However, the combined impact of patient size, tube voltage, and heart rate (HR) on VMI of coronary CT angiography (CCTA) remains unknown. This phantom study aimed to assess VMI levels achieving comparable contrast-to-noise ratio (CNR) in CCTA at 50% CM dose across varying tube voltages, patient sizes, and HR, compared to the reference protocol (100% CM dose, conventional at 120 kVp). A 5 mm artificial coronary artery with 100% (400 HU) and 50% (200 HU) iodine CM-dose was positioned centrally in an anthropomorphic thorax phantom. Horizontal coronary movement was matched to HR (at 0, < 60, 60–75, > 75 bpm), with varying patient sizes simulated using phantom extension rings. Raw data was acquired using a clinical CCTA protocol at 120 and 140 kVp (five repetitions). VMI images (40–70 keV, 5 keV steps) were then reconstructed; non-overlapping 95% CNR confidence intervals indicated significant differences from the reference. Higher CM-dose, reduced VMI, slower HR, higher tube voltage, and smaller patient sizes demonstrated a trend of higher CNR. Regardless of HR, patient size, and tube voltage, no significant CNR differences were found compared to the reference, with 100% CM dose at 60 keV, or 50% CM dose at 40 keV. DLCT reconstructions at 40 keV from 120 to 140 kVp acquisitions facilitate 50% CM dose reduction for various patient sizes and HR with equivalent CNR to conventional CCTA at 100% CM dose, although clinical validation is needed.

**Supplementary Information:**

The online version contains supplementary material available at 10.1007/s10554-024-03062-6.

## Introduction

Coronary computed tomography angiography (CCTA) has emerged as the clinical diagnosis standard for coronary artery disease (CAD) in patients with stable chest pain [1]. Additionally, CCTA is recommended by the European Society of Cardiology (ESC) for the screening of chronic coronary syndrome in patients with an intermediate risk of CAD [2–4]. This non-invasive imaging modality has gained considerable popularity due to its very good sensitivity and negative predictive value (85–91% and 83–91%, respectively) for detecting coronary plaque and ruling out significant stenosis [5–7]. Moreover, emerging technologies in CCTA have the potential to facilitate personalized risk assessment through plaque characterization and cardiovascular metrics [8]. Bridging the gap between these advancements and clinical application warrants careful consideration of the drawbacks of iodinated contrast medium (CM) administration against the benefit of CCTA. Although the incidence of post-contrast acute kidney injury (PC-AKI) in patients is controversial, patients with CAD often exhibit risk factors for PC-AKI, such as estimated glomerular filtration rate (eGFR) < 30 ml/min/1.73 m^2^, severe heart disease, diabetes mellitus, dehydration, and use of nephrotoxic medication [9–11]. Additionally, CM contributes to an increasing worldwide environmental risk by contamination of drinking water sources [12]. Decreasing CM dose combines goals for reducing patient risk, costs, and the ecological footprint associated with pharmaceutical waste. Therefore, the most recent guidelines of the Society of Cardiovascular Computed Tomography (SCCT) recommend reduced tube voltage acquisitions or dual-energy acquisitions with virtual mono-energetic image (VMI) reconstructions for CCTA examinations [13].

One of the available dual-energy or spectral imaging techniques is detector-based dual-layer CT (DLCT), which enables VMI reconstructions across a wide energy spectrum ranging from 40 to 200 kiloelectron volt (keV). Attenuation of iodinated CM increases at low energy levels (< 70 keV), approaching the k-edge of iodine at 33.2 keV, and thereby improving vascular enhancement through the photoelectric effect. Van Hamersvelt et al. demonstrated a potential reduction in CM dose by 40–60% at maintained image quality through the utilization of low VMI reconstructions in-vitro [14]. This has been verified by several other phantom and clinical studies [15–19].

By lowering the tube voltage from 140 to 120 kilovolt peak (kVp), vascular contrast enhancement is increased by 20–25% [20, 21]. Besides facilitating CM reduction, the use of lower tube voltages also results in a reduction of radiation dose [14, 19, 22]. However, obtaining diagnostic images in obese patients may be challenging, as the presence of increased adipose tissue leads to higher photon attenuation, adversely affecting contrast-to-noise ratios (CNR) and noise levels [23]. Therefore, patient size must be taken into careful consideration when considering optimal VMI levels and tube voltages for diagnostic cardiac imaging.

Similarly, elevated heart rate (HR) is a known cause of motion artifacts and reduced image quality in CCTA. Despite the potential benefits of CM dose reduction and the impact of patient characteristics on image quality, to the best of our knowledge, no study has systematically evaluated the combined impact of patient size, tube voltage, and HR on CM dose reduction for VMI reconstructions [16]. These findings may guide clinicians in tailoring imaging protocols based on indivdual patient characteristics, thereby optimizing both diagnostic accuracy and patient safety.

Therefore, our aim was to systematically assess VMI levels that achieve comparable CNR for DLCT at 50% CM-dose at different tube voltages, simulated patient sizes, and HR compared to the reference protocol (120 kVp, conventional reconstruction, 100% CM dose).

## Materials and methods

### Phantom design and setup

**Fig. 1:**
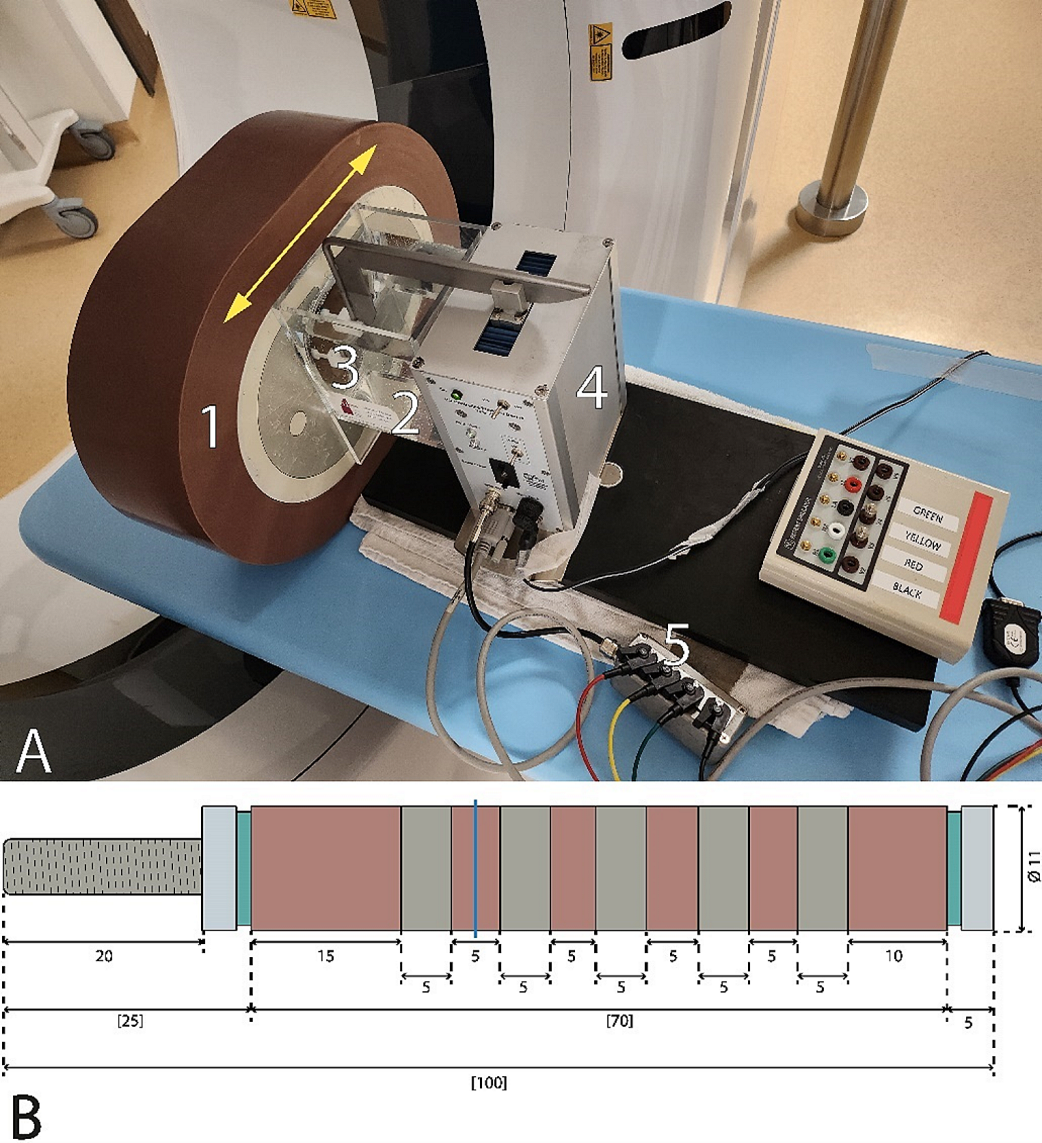
Overview anthropomorphic thorax phantom. **a**) An anthropomorphic thorax phantom was used to simulate three patient sizes using various extension rings of fat−equivalent material (1). The phantom included a fillable water compartment (2) with a hollow artificial coronary artery (3) filled with iodinated contrast material at different dilutions. A computer−controlled motion unit (4) moved the artificial artery at various velocities along the direction indicated by the yellow arrow. The velocities correspond to different heart rates, and the image acquisition was synchronized to the robotic arm movement cycle by using a simulated electrocardiogram (ECG) signal output (5) of the motion unit as ECG input for the CT. **b**) The artificial artery contains solid water sections (red), and five hydroxyapatite calcifications (grey). All measurements were conducted in the middle of the solid water region between the two leftmost calcification rings (blue line). At the center of the artificial artery, a lumen of 5 mm in diameter was present throughout the phantom, which was filled with two contrast media concentrations

An anthropomorphic thorax phantom (QRM-Thorax, PTW GmbH) consisting of a spine insert, artificial lungs, and soft tissue-specific material was used for this study. Three patient sizes were simulated using two fatty tissue-specific extension rings (QRM-Extension Ring M and L, PTW GmbH) to increase the outer diameter of the phantom model from 300 × 200 mm with no ring (small patient size) to 350 × 250 mm with a medium ring (average patient size) and 400 × 300 mm with a large ring (large patient size), Fig. 1a [24].

At the center of the thorax phantom, we positioned a water compartment containing a hollow artificial coronary artery of 5 mm diameter, Fig. 1b. The lumen of this artificial artery was filled with iodinated CM (Omnipaque 350; GE Healthcare) at two different dosages, resulting in an approximate attenuation of 400 and 200 Hounsfield units (HU) at 100% and 50% CM dose at 120 kVp and a small patient size, respectively [25–27]. A computer-controlled motion unit (QRM-Sim2D, PTW GmbH) was used to move the artificial coronary artery within the water-filled compartment in the horizontal plane perpendicular to the scan direction at constant velocities of 0, 10, 20, and 30 mm/s, which are average in-vivo coronary movement velocities at HR of 0, < 60, 60–75, and > 75 beats per minute (bpm), respectively [28]. To ensure linear motion, image acquisition was synchronized to the robotic arm movement cycle by using a simulated electrocardiogram (ECG) signal output of the motion unit as ECG input for the CT.

### Data acquisition and reconstruction

All data was acquired on a DLCT (Spectral CT7500, Philips Healthcare) using a clinical protocol for CCTA, Table 1. In line with vendor recommendations, a sequential scan mode was used for HR of 0, < 60, and 60–75 bpm, while a low pitch helical acquisition with retrospective gating was performed for > 75 bpm. Each axial data acquisition covered a 360° angular range and offered a z-coverage of the reconstruction volume just under the 8 cm detector width. Image reconstruction was performed using an aperture-weighted cone beam approach with a slice thickness of 0.9 mm and an increment of 0.45 mm. For each combination of HR and patient size, two parameters were adjusted: tube voltage (120 and 140 kVp) and CM dose (100% and 50%). For each of these combinations of HR and patient size, the conventional acquisition at 120 kVp with 100% CM dose was defined as the reference. All scans with 50% CM dose were defined as experimental to improve readibility. The phantom was scanned five times for each combination of CM dose, tube voltage, HR, and patient size, with a small translation and rotation of the phantom between each scan. In addition to the conventional reconstruction, VMI levels were reconstructed from 40 to 70 keV at intervals of 5 keV.

**Table 1:** Acquisitions and reconstruction parameters

Philips CT 7500	Sequential		Helical	
Tube voltage, kVp	120	140	120	140
mAs	110	75	340	235
CTDI, mGy	4.6	5.6	22.4	22.6
Collimation, mm	128 x 0.625		128 x 0.625	
Rotation time, ms	270		270	
Matrix	512 x 512		512 x 512	
Phase, %	75		75	
Slice thickness, mm	0.90		0.90	
Pitch	n/a		0.16	
Increment, mm	0.45		0.45	
Iterative reconstruction	IMR1		IMR1	
Image definition	Cardiac Routine		Cardiac Routine	
DRI	16		30	

According to clinical protocols, the sequential (prospective ECG−triggered) acquisition was used for all heart rates <75 beats per minute (bpm), while the helical (retrospective ECG−gated) scan mode was used for >75 bpm

Abbreviations: bpm: beats per minute, CTDI: computed tomography dose index, DRI: dose right index (image quality reference setting), ECG: electrocardiogram, IMR: iterative model reconstruction, kVp: kilovolt peak, mGy: milligray, mm: millimeter, ms: milliseconds

### Data analysis

**Fig. 2:**
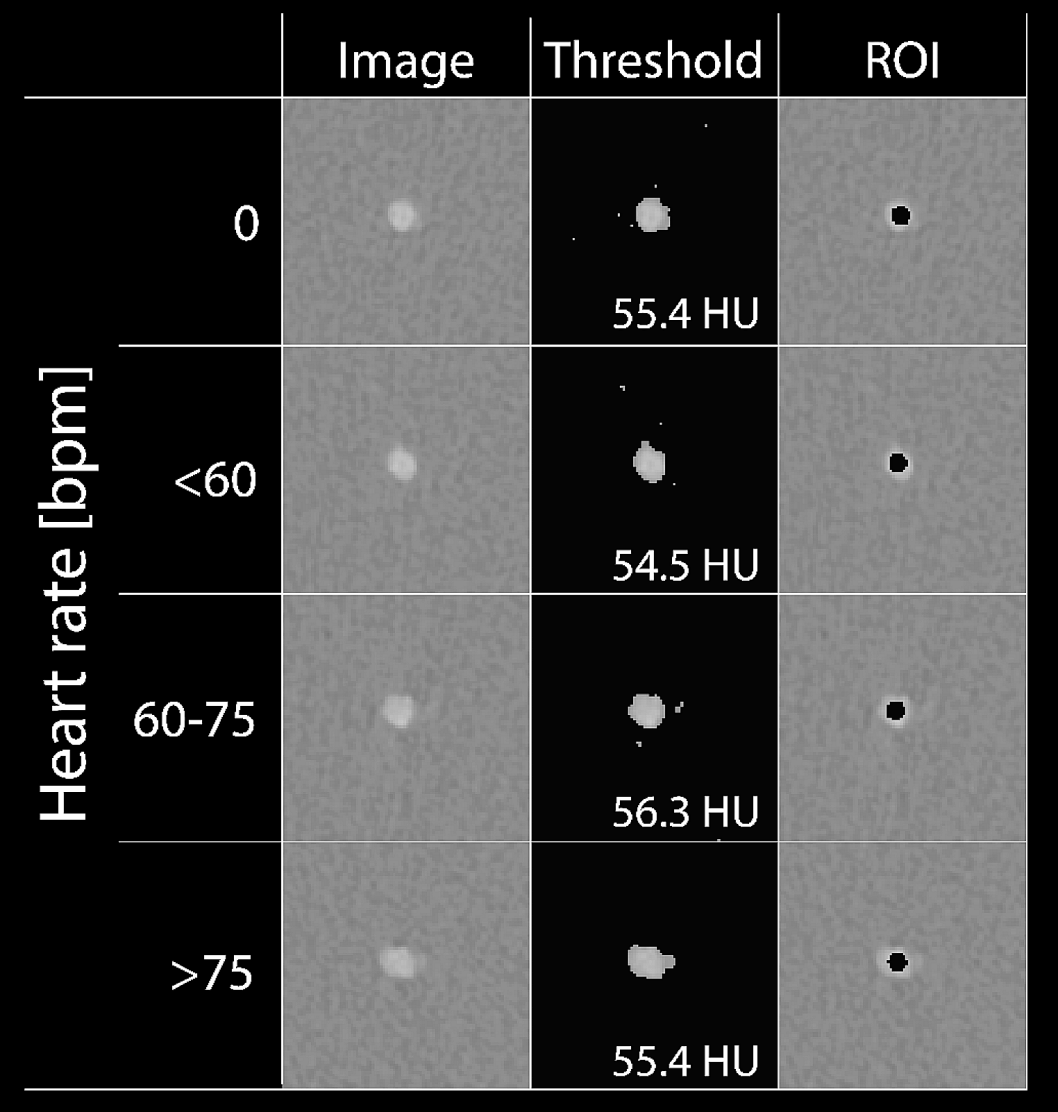
Summary of Image analysis. The threshold application on the original data (left, window setting: 750/90 HU (W/L)) is used to discriminate between background material (water) and contrast material (CM) in the lumen using a fully automatically calculated threshold based on the mean and standard deviation (SD) of the background signal (threshold = mean background + 3.5 times SD background) (middle). For each heart rate, the threshold in Hounsfield Units (HU) is indicated. The resulting contrast−to−noise ratio was based on the attenuation difference between the background and lumen, whereby the lumen attenuation was calculated within 60% of the total lumen (indicated in black in the rightmost column), to omit any edge or partial volume effects. Abbreviations: bpm: beats per minute, CM: contrast medium, HU: Hounsfield units, SD: standard deviation

To assess the added value of reduced VMI levels, CNR was assessed from all reconstructions using a validated Python script (version 3.8) [29]. Measurements were exclusively confined to the solid water section of the phantom, specifically the most centrally located slice between the two leftmost and least dense calcification rings, Fig. 1b. The mean and standard deviation (SD) was calculated in a ROI at the center of the lumen, Fig. 2. A simple threshold was used to discriminate background material (water) from CM in the lumen. The value of the threshold was the mean of the background signal plus 3.5 times the SD.

CNR was defined as:

$$\text{C}\text{N}\text{R}=\frac{{\text{C}\text{T}}_{\text{l}\text{u}\text{m}\text{e}\text{n}}-{\text{C}\text{T}}_{\text{b}\text{a}\text{c}\text{k}\text{g}\text{r}\text{o}\text{u}\text{n}\text{d}}}{{{\upsigma }}_{\text{b}\text{a}\text{c}\text{k}\text{g}\text{r}\text{o}\text{u}\text{n}\text{d}}}$$

with CT_lumen_ the mean CT number (in HU) of the coronary artery, CT_background_ the mean CT number of the background material, and σ_background_ the standard deviation of the mean HU of the background. The lumen was defined as a central ROI of 60% of the total lumen area to omit any edge effects, Fig. 2. For all acquisitions, CNR with 95% confidence intervals (CI) were calculated from the five repetitions for each patient size, HR, tube voltage, and CM dose. For each combination of patient size and HR, CNR was compared to the reference. Non-overlapping 95% CI of CNR were deemed significantly different. For each patient size, noise was compared to the reference (conventional reconstruction at 120 kVp, 100% CM dose, and at 0 bpm). Statistical analysis was performed with SPSS v26.0 (IBM SPSS version 26.0, IBM corporation).

## Results

At the reference, the mean luminal CM attenuation [95% CI] was 411 [400, 423], 393 [374, 413], and 318 [281, 356] HU for small, medium, and large phantoms, respectively. At 50% CM, the corresponding luminal attenuation was 192 [166, 219], 201 [194, 207], and 193 [185, 202] HU. Attenuation, noise and CNR for all combinations of CM doses, tube voltages, VMI levels, patient sizes and HR are shown in Online Resource 1–4. Volumetric CT dose index volume (CTDIvol) values are listed in Table 1.

### Attenuation

**Fig. 3:**
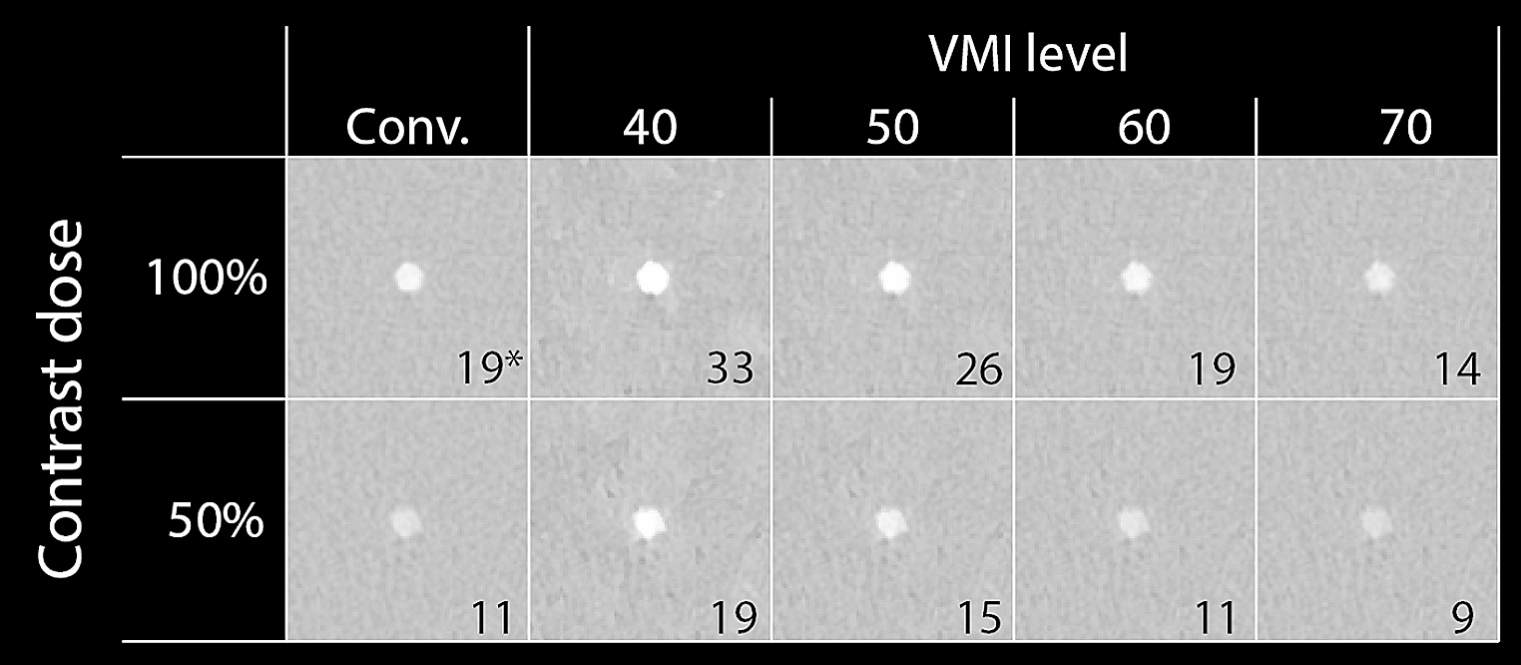
Representative images of 100% and 50% contrast dose. The conventional (Conv.) image at 120 kVp and four virtual monoenergetic image (VMI) levels (70/90 HU (W/L)) are provided for a static lumen and large patient size. The reference result is indicated with an asterisk*. For all reconstructions, the resulting contrast−to−noise−ratio) is given in the right lower corner. Abbreviations: VMI: virtual monoenergetic image, Conv.: conventional, keV: kiloelectron volt, kVp: kilovolt peak

With decreasing VMI levels, attenuation increased for all patient sizes, HR, CM dilutions and tube voltage. For all experimental acquisitions (50% CM dose) at least one VMI level showed comparable attenuation to the reference. At 100% CM dose, a VMI at 60 keV showed the least difference in attenuation compared to conventional acquisition (Fig. 3).

### Noise

The mean noise level of the reference was 12 [11, 12], 15 [15, 16] and 19 [19, 20] HU for small, medium, and large patients, respectively. Mean noise was lower at 140 kVp and all VMI levels (Online Resource 1–4). VMI showed increasing degrees of noise with decreasing VMI levels for all patient sizes and HR, and for both contrast dosages and tube voltages. Experimental acquisitions at 40 keV showed higher mean noise than the reference, with experimental VMI at 65 keV possessing the most comparable noise levels to the reference.

### Contrast-to-noise ratio

**Fig. 4:**
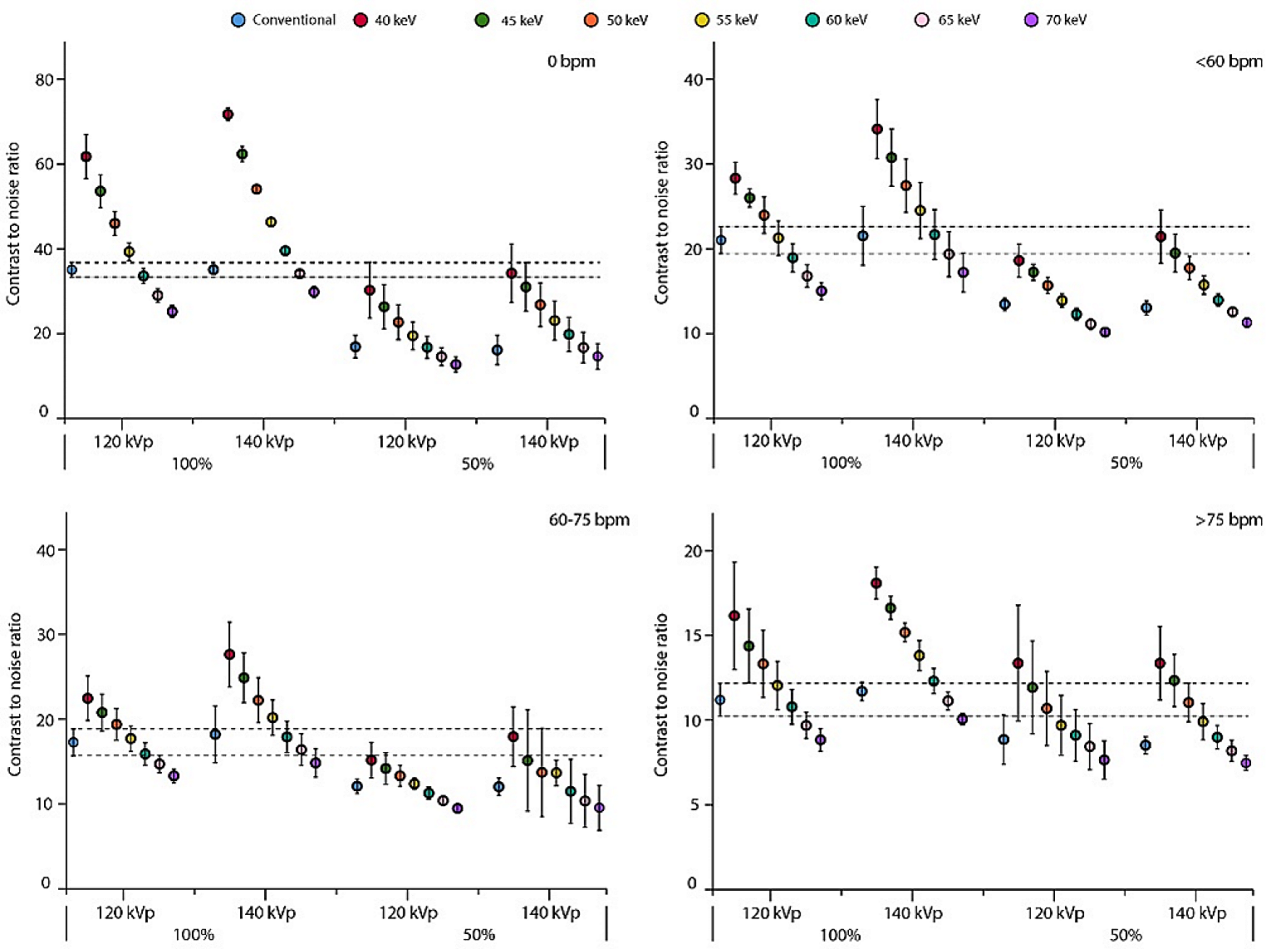
Graph of contrast-to-noise ratios (CNR) for a small patient size. Data are presented as mean and 95% confidence interval. The reference (conventional reconstruction, 120 kVp, 100% contrast dose) confidence interval is extrapolated with dotted line, to assess significant differences for all other combinations of tube voltage, contrast dose, and reconstruction. Results are shown for simulated heart rates of 0 (top left), <60 (top right), 60–75 (bottom left), and >75 (bottom right) beats per minute. Abbreviations: bpm: beats per minute, keV: kiloelectron volt, kVp: kilovolt peak

**Fig. 5:**
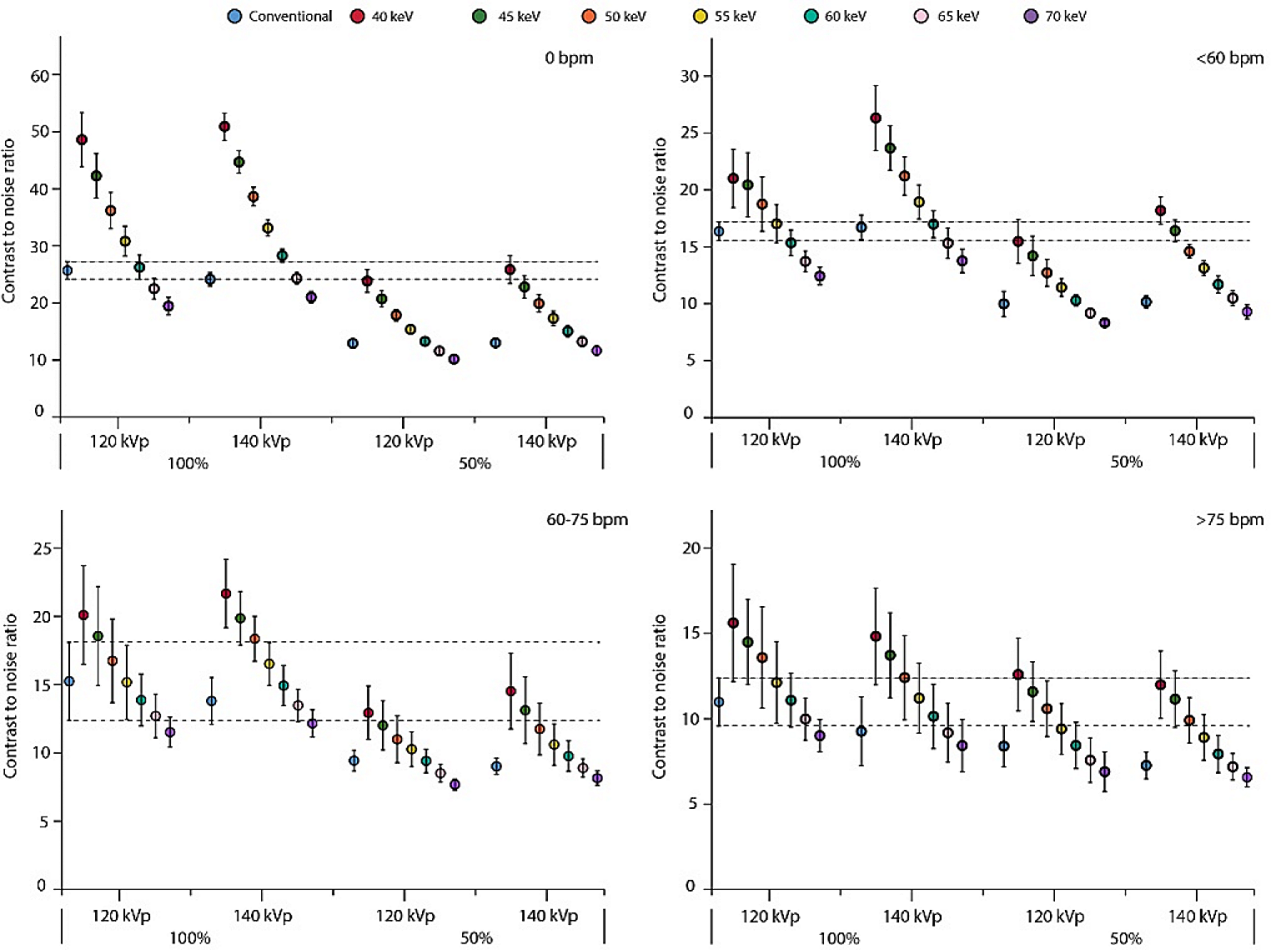
Contrast-to-noise ratios for an average patient size. Data are presented as mean and 95% confidence interval. The reference (conventional reconstruction, 120 kVp, 100% contrast dose) confidence interval is extrapolated with dotted line, to assess significant differences for all other combinations of tube voltage, contrast dose, and reconstruction. Results are shown for heart rates of 0 (top left), <60 (top right), 60–75 (bottom left), and >75 (bottom right) beats per minute. Abbreviations: bpm: beats per minute, keV: kiloelectron volt, kVp: kilovolt peak

**Fig. 6:**
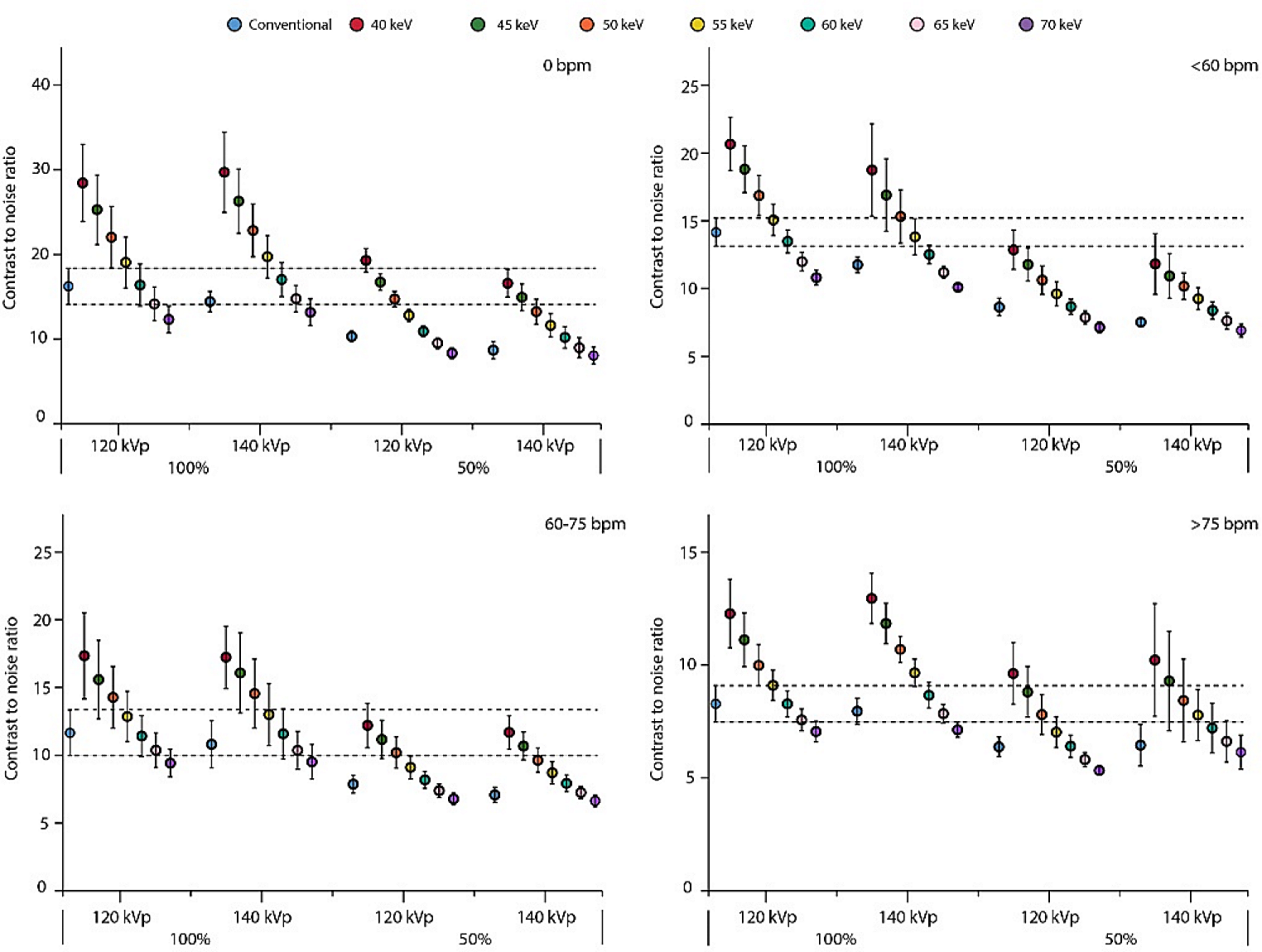
Contrast-to-noise ratios for a large patient size. Data are presented as mean and 95% confidence interval. The reference (conventional reconstruction, 120 kVp, 100% contrast dose) confidence interval is extrapolated with dotted line, to assess significant differences for all other combinations of tube voltage, contrast dose, and reconstruction. Results are shown for heart rates of 0 (top left), <60 (top right), 60–75 (bottom left), and >75 (bottom right) beats per minute. Abbreviations: mAs: milliampere−seconds, bpm: beats per minute, keV: kiloelectron volt, kVp: kilovolt peak

VMI showed increasing CNR with decreasing VMI levels for all patient sizes and HR, and for both contrast dosages and tube voltages (Figs. 4, 5 and 6). For all experimental acquisitions (50% CM dose), at least one VMI level showed comparable CNR to the reference, ranging from 40 to 65 keV, Table 2. VMI from 140 kVp tended to show slightly higher CNR than from 120 kVp, but not for large patients or HR > 75 bpm, although the differences were not significant (Figs. 4, 5 and 6). At 100% CM dose, VMI at 60 keV showed the least difference in CNR compared to the reference. For acquisitions < 60 bpm at 120 kVp with 50% CM dose, comparable CNR were found at VMI levels of 40, 40–45 and 40 keV for small, medium, and large patients, respectively (Table 2). As the HR increased to 60–75 bpm, those VMI levels shifted to 40–45 for small patients, 40–45 for medium patients, and 40–50 keV for large patients. Similarly, for HR > 75 bpm, comparable CNR were found at VMI levels of 40–60, 40–60, and 40–55 keV for small, medium, and large patients, respectively.

**Table 2:** keV equivalent (range) compared to the heart rate category specific conventional reference (120 kVp; 100% contrast medium) per stratification

kVp level	CM dose	Small				Medium				Large			
		0 bpm	< 60 bpm	60–75 bpm	> 75 bpm	0 bpm	< 60 bpm	60–75 bpm	> 75 bpm	0 bpm	< 60 bpm	60–75 bpm	> 75 bpm
120	100%	60	50–60	40–60	50–65	60	50–60	40–70	40–70	55–65	55–60	45–70	55–70
140	100%	65	55–70	55–70	60–70	60–65	60–65	45–70	40–75	55–70	45–60	45–70	55–65
120	50%	40	40	40–45	40–60	40	40–45	40–45	40–60	40–50	40	40–50	40–55
140	50%	40–45	40–45	40–50	40–55	40–45	40–45	40–50	40–55	40–50	40	40–50	40–65

Abbreviations: CM: contrast medium, bpm: beats per minute, keV: kiloelectron volt, kVp: kilovolt peak

## Discussion

This phantom study assessed the CNR in a reduced CM dose dual-energy CCTA protocol, considering different simulated patient sizes, VMI levels, kVp settings, and HR. Notably, a VMI level of 40 keV facilitated CM dose savings of up to 50% with comparable CNR for all combinations of the evaluated HR and patient sizes.

Additionally, we observed a consistent increase in CNR with decreasing VMI levels for all acquisitions. This indicates that the attenuation of iodine increases relatively more than background noise. However, it is essential to recognize that this effect may vary between different scanner vendors, necessitating protocol optimization for individual scanners [14].

Our findings align well with previous clinical and phantom studies that demonstrated similar CM reduction in dual-energy CCTA while maintaining sustained CNR [16, 30–33]. Oda et al. studied the effects of VMI using a DLCT to reduce the CM dose by 50% in 60 patients referred for CCTA [30]. Notably, 50% of the included patients had renal insufficiency. The authors reported a significant increase in mean coronary attenuation and visual scores, while no significant differences were noted in other aspects of visual image quality at a VMI of 50 keV when compared to conventional 120 kVp. Similarly, Raju et al. included 102 patients who were randomized to conventional CCTA (*n* = 53) and received 80 ml CM (120 kVp if BMI > 30, 100 kVp if BMI < 30), or fast kVp-switching dual-energy computed tomography (DECT) (*n* = 49) using 35 ml CM and 60 keV VMI reconstruction. The study reported a comparable signal-to-noise ratio, CNR, and rate of diagnostic interpretability, but inferior image quality Likert scores of the DECT cohort [33]. It is unclear if the > 50% contrast reduction combined with a relatively high VMI of 60 keV might have affected these inferior image quality scores. To the best of our knowledge, no previous clinical studies have stratified patients based on patient size or HR, and no phantom studies have incorporated a moving coronary artery to simulate different HR. Thus, our phantom study provides novel insights, suggesting that dual-energy CCTA with half CM dose at low VMI (40 keV) reconstruction show comparable image quality to conventional CCTA in most settings. In clinical practice, the potential for CM reduction is limited in some patient groups as a VMI level of 70 keV or higher is considered optimal for the evaluation of plaques and late enhancement, or in the precense of stents and implants. Specifically, our study did not incorporate cardiovascular disease, and, therefore, whether 40 keV provides sufficient image quality in intermediate risk patients with low calcium scores, requires further investigation.

Dual-energy acquisitions at higher tube voltages are expected to increase penetration of high-energy photons into the lower detector layer and improve spectral separation and sensitivity [34, 35]. Although few studies have compared dual-energy CCTA at different tube voltages, acquisitions at 140 kVp have demonstrated higher agreement with invasive coronary angiograms [36], albeit at the cost of a higher radiation dose. Similarly, a reduced 100 kVp CCTA protocol has been proposed to improve iodine attenuation, enabling both CM dose and radiation dose reduction [37]. The PROTECTION VI study reported a median radiation dose reduction of 50%, and of CM volume by 13% for tube voltages of 90–100 kVp when compared to conventional 120 kVp imaging. However, at the time of data acquisition of this study, our scanner could not acquire spectral data at 100 kVp. Consequently, further research initiatives are needed to evaluate the effects of additional tube voltage reduction.

Our study presents a potential for substantially reducing CM dose, thereby potentially mitigating health impact on patients. Moreover, given that via urine excretion CM enters the ecosystem, elevated CM levels have been detected in drinking water in several countries, therefore moderating CM usage is becoming ever more relevant [12]. Although CM are metabolically stable, they have shown to be degradable, reactive, and unstable under normal environmental conditions, posing potentially unwanted and unexpected ecological effects [38].

Several limitations may have influenced the results obtained in this study. First, the linear two-dimensional motion of the phantom in this study does not completely simulate a clinical situation, as in-vivo movements are three dimensional, and much more complex. In addition, the low VMI reconstructions used in this study might also affect subjective image quality by introducing blooming artifacts, for example in segments with calcifications. As the current study utilized no cardiovascular disease, future studies should be leveraged in clinical practice. Second, HR were translated and approximated to motion velocities based on one single study [28]. However, the broad HR ranges used in this study warrant this limitation. Third, due to inherent vendor-specific differences, our results may not be valid for other scanners. Finally, it is noteworthy that, lowering VMI levels may introduce artifacts, thus the CNR reported in this study might not be fully representative for the image quality as a whole in a clinical setting and clinical testing will be required before implementation of low contrast protocols.

## Conclusion

This comprehensive phantom study indicated that a detector-based DLCT image reconstruction at 40 keV may facilitate 50% CM dose reduction for various patient sizes and HR with equivalent CNR compared to conventional CCTA at 100% CM dose, although clinical validation is needed.

### Electronic supplementary material

Below is the link to the electronic supplementary material.

Supplementary Material 1

Supplementary Material 2

Supplementary Material 3

Supplementary Material 4

Supplementary Material 5

## Data Availability

No datasets were generated or analysed during the current study.

## Abbreviations

bpm: Beats per minute
CAD: Coronary artery disease
CCTA: Coronary computed tomography angiography
CI: Confidence interval
CM: Contrast medium
CNR: Contrast–to–noise ratio
CTDI: Computed tomography dose index
DECT: Dual–energy computed tomography
DLCT: Dual–layer computed tomography
ECG: Electrocardiogram
eGFR: Estimated global filtration rate
HR: Heart rate
HU: Hounsfield units
IMR: Iterative model reconstruction
keV: Kiloelectron volt
kVp: Kilovolt peak
mAs: Milliampere–seconds
ms: Milliseconds
PC-AKI: Post–contrast acute kidney injury
ROI: Region of interest
SD: Standard deviation
VMI: Virtual mono–energetic image

## References

[1] Moss AJ, Williams MC, Newby DE, Nicol ED (2017). The updated NICE guidelines: cardiac CT as the First-Line Test for Coronary Artery Disease. Curr Cardiovasc Imaging Rep.

[2] Saraste A, Barbato E, Capodanno D (2019). Imaging in ESC clinical guidelines: chronic coronary syndromes. Eur Heart J Cardiovasc Imaging.

[3] Newby DE, Adamson PD, Berry C (2018). Coronary CT angiography and 5-Year risk of myocardial infarction. N Engl J Med.

[4] Knuuti J, Wijns W, Saraste A (2020). 2019 ESC guidelines for the diagnosis and management of chronic coronary syndromes. Eur Heart J.

[5] Weir-McCall JR, Williams MC, Shah ASV (2023). National trends in Coronary Artery Disease Imaging. JACC Cardiovasc Imaging.

[6] Miller JM, Rochitte CE, Dewey M (2008). Diagnostic performance of coronary angiography by 64-Row CT. N Engl J Med.

[7] Neglia D, Rovai D, Caselli C et al (2015) Detection of significant coronary artery disease by Noninvasive Anatomical and Functional Imaging. Circ Cardiovasc Imaging 8. 10.1161/CIRCIMAGING.114.002179 10.1161/CIRCIMAGING.114.002179 25711274

[8] Abdelrahman KM, Chen MY, Dey AK (2020). Coronary computed tomography angiography from clinical uses to Emerging technologies. J Am Coll Cardiol.

[9] Davenport MS, Perazella MA, Yee J (2020). Use of Intravenous Iodinated contrast media in patients with kidney disease: Consensus statements from the American College of Radiology and the National Kidney Foundation. Radiology.

[10] van der Molen AJ, Reimer P, Dekkers IA (2018). Post-contrast acute kidney injury – part 1: definition, clinical features, incidence, role of contrast medium and risk factors. Eur Radiol.

[11] van der Molen AJ, Reimer P, Dekkers IA (2018). Post-contrast acute kidney injury. Part 2: risk stratification, role of hydration and other prophylactic measures, patients taking metformin and chronic dialysis patients. Eur Radiol.

[12] Dekker HM, Stroomberg GJ, Prokop M (2022). Tackling the increasing contamination of the water supply by iodinated contrast media. Insights Imaging.

[13] Abbara S, Blanke P, Maroules CD (2016). SCCT guidelines for the performance and acquisition of coronary computed tomographic angiography: a report of the Society of Cardiovascular Computed Tomography Guidelines Committee. J Cardiovasc Comput Tomogr.

[14] van Hamersvelt RW, Eijsvoogel NG, Mihl C (2018). Contrast agent concentration optimization in CTA using low tube voltage and dual-energy CT in multiple vendors: a phantom study. Int J Cardiovasc Imaging.

[15] Jin L, Jie B, Gao Y, et al (2021) Low dose contrast media in step-and-shoot coronary angiography with third-generation dual-source computed tomography: feasibility of using 30 mL of contrast media in patients with body surface area &lt;1.7 m2. Quant Imaging Med Surg 11:2598–2609. 10.21037/qims-20-500 10.21037/qims-20-500 PMC8107323 34079726

[16] Rotzinger DC, Si-Mohamed SA, Yerly J (2021). Reduced-iodine-dose dual-energy coronary CT angiography: qualitative and quantitative comparison between virtual monochromatic and polychromatic CT images. Eur Radiol.

[17] Yoshida R, Usui K, Katsunuma Y (2020). Reducing contrast dose using virtual monoenergetic imaging for aortic CTA. J Appl Clin Med Phys.

[18] Mahmoudi S, Lange M, Lenga L et al (2022) Salvaging low contrast abdominal CT studies using noise-optimised virtual monoenergetic image reconstruction. 10.1259/bjro.20220006. BJR|Open 4:10.1259/bjro.20220006 PMC9446156 36105416

[19] Leng S, Yu L, Fletcher JG, McCollough CH (2015). Maximizing iodine contrast-to-noise ratios in abdominal CT imaging through Use of Energy Domain noise reduction and virtual Monoenergetic Dual-Energy CT. Radiology.

[20] Fleischmann D, Chin AS, Molvin L (2016). Computed tomography angiography. Radiol Clin North Am.

[21] Hallett RL, Molvin L, Fleischmann D (2020) CT angiography technique: contrast Medium Dynamics. Low- Tube-Voltage, and Dual-Energy Imaging

[22] Lee AM, Engel L-C, Hui GC (2014). Coronary computed tomography angiography at 140 kV *versus* 120 kV: assessment of image quality and radiation exposure in overweight and moderately obese patients. Acta Radiol.

[23] Kang H-J, Lee JM, Lee SM (2019). Value of virtual monochromatic spectral image of dual-layer spectral detector CT with noise reduction algorithm for image quality improvement in obese simulated body phantom. BMC Med Imaging.

[24] McCollough CH, Ulzheimer S, Halliburton SS (2007). Coronary artery calcium: a multi-institutional, Multimanufacturer International Standard for Quantification at Cardiac CT. Radiology.

[25] Fei X, Du X, Yang Q (2008). 64-MDCT coronary angiography: Phantom Study of effects of Vascular attenuation on detection of coronary stenosis. Am J Roentgenol.

[26] Cademartiri F, Maffei E, Palumbo AA (2008). Influence of intra-coronary enhancement on diagnostic accuracy with 64-slice CT coronary angiography. Eur Radiol.

[27] Utsunomiya D, Tanaka R, Yoshioka K (2016). Relationship between diverse patient body size- and image acquisition-related factors, and quantitative and qualitative image quality in coronary computed tomography angiography: a multicenter observational study. Jpn J Radiol.

[28] Husmann L, Leschka S, Desbiolles L (2007). Coronary artery motion and Cardiac Phases: dependency on Heart Rate—implications for CT Image Reconstruction. Radiology.

[29] van Praagh GD, van der Werf NR, Wang J (2021). Fully automated quantification method (FQM) of coronary calcium in an anthropomorphic phantom. Med Phys.

[30] Oda S, Takaoka H, Katahira K (2019). Low contrast material dose coronary computed tomographic angiography using a dual-layer spectral detector system in patients at risk for contrast-induced nephropathy. Br J Radiol.

[31] Huang X, Gao S, Ma Y (2020). The optimal monoenergetic spectral image level of coronary computed tomography (CT) angiography on a dual-layer spectral detector CT with half-dose contrast media. Quant Imaging Med Surg.

[32] Yi Y, Zhao X-M, Wu R-Z (2019). Low dose and low contrast medium coronary CT angiography using dual-layer spectral detector CT. Int Heart J.

[33] Raju R, Thompson AG, Lee K (2014). Reduced iodine load with CT coronary angiography using dual-energy imaging: a prospective randomized trial compared with standard coronary CT angiography. J Cardiovasc Comput Tomogr.

[34] Pelgrim GJ, van Hamersvelt RW, Willemink MJ (2017). Accuracy of iodine quantification using dual energy CT in latest generation dual source and dual layer CT. Eur Radiol.

[35] Borges AP, Antunes C, Curvo-Semedo L (2023). Pros and cons of dual-energy CT systems: one does not fit all. Tomography.

[36] Pelter M, Almeida S, Bagsic S (2022). 424 image quality, Radiation Dosimetry, and diagnostic accuracy of whole heart single heartbeat coronary ct angiography as validated by invasive coronary angiogram in a high calcium score Population. J Cardiovasc Comput Tomogr.

[37] Stocker TJ, Leipsic J, Hadamitzky M (2020). Application of low tube potentials in CCTA. JACC Cardiovasc Imaging.

[38] Plewa MJ, Wagner ED, Richardson SD (2004). Chemical and Biological characterization of newly discovered Iodoacid drinking Water Disinfection byproducts. Environ Sci Technol.

