# Supplementary material for: Halved contrast medium dose coronary dual-layer CT-angiography – phantom study of tube current and patient characteristics
Source: Int J Cardiovasc Imaging. 2024 Feb 22;40(4):931–40. doi: 10.1007/s10554-024-03062-6 (PMC11052773; doi:10.1007/s10554-024-03062-6)
Supplement: Supplementary file 5 — Supplementary Material 5 [file 10554_2024_3062_MOESM5_ESM.docx]

**Supplementary**

|  | | | **Full** | | | | | | | |
| --- | --- | --- | --- | --- | --- | --- | --- | --- | --- | --- |
|  |  |  | **120 kVp** | | | | | | | |
|  |  |  | **Conv** | **40 keV** | **45 keV** | **50 keV** | **55 keV** | **60 keV** | **65 keV** | **70 keV** |
| **Small** | **0 bpm** | **CNR** | 25 [24-26] | 33 [30-36] | 30 [28-32] | 26 [25-28] | 23 [22-24] | 21 [20-21] | 18 [18-19] | 16 [16-17] |
|  |  | **Attenuation** | 411 [404-420] | 964 [933-995] | 773 [750-796] | 627 [610-644] | 515 [502-528] | 429 [419-439] | 362 [353-372] | 310 [302-319] |
|  |  | **Noise** | 12 [11-12] | 15 [14-16] | 14 [13-15] | 13 [13-14] | 13 [13-13] | 13 [12-13] | 13 [12-13] | 12 [12-13] |
|  | **<60 bpm** | **CNR** | 21 [20-22] | 29 [27-30] | 26 [25-27] | 24 [23-26] | 22 [20-23] | 19 [18-20] | 17 [16-18] | 15 [14-16] |
|  |  | **Attenuation** | 256 [242-271] | 445 [417-473] | 378 [363-393] | 329 [310-349] | 281 [264-298] | 245 [230-260] | 213 [201-225] | 189 [178-199] |
|  |  | **Noise** | 12 [12-12] | 16 [15-16] | 14 [14-15] | 14 [13-14] | 13 [13-14] | 13 [12-13] | 13 [12-13] | 13 [12-13] |
|  | **60-75 bpm** | **CNR** | 18 [16-19] | 23 [21-25] | 21 [20-23] | 20 [18-21] | 18 [17-19] | 16 [15-17] | 15 [14-16] | 13 [13-14] |
|  |  | **Attenuation** | 203 [187-218] | 348 [304-392] | 295 [262-329] | 259 [233-284] | 226 [207-246] | 197 [181-214] | 178 [163-193] | 159 [147-172] |
|  |  | **Noise** | 12 [11-12] | 15 [15-16] | 14 [13-15] | 13 [13-14] | 13 [12-13] | 12 [12-13] | 12 [12-13] | 12 [11-12] |
|  | **>75 bpm** | **CNR** | 11 [10-12] | 16 [14-18] | 14 [13-16] | 13 [12-15] | 12 [11-13] | 11 [10-12] | 10 [9-10] | 9 [8-9] |
|  |  | **Attenuation** | 128 [122-134] | 246 [234-258] | 205 [197-213] | 178 [170-186] | 155 [148-161] | 135 [130-139] | 118 [115-121] | 106 [104-108] |
|  |  | **Noise** | 11 [11-12] | 15 [13-17] | 14 [13-15] | 13 [12-14] | 13 [12-14] | 12 [12-13] | 12 [12-13] | 12 [11-13] |
| **Medium** | **0 bpm** | **CNR** | 18 [18-19] | 29 [27-31] | 25 [24-27] | 22 [21-24] | 20 [19-21] | 17 [16-18] | 15 [14-16] | 13 [13-14] |
|  |  | **Attenuation** | 393 [379-407] | 962 [918-1007] | 771 [736-807] | 625 [597-653] | 513 [490-536] | 427 [408-446] | 360 [344-376] | 308 [294-322] |
|  |  | **Noise** | 16 [15-16] | 22 [21-23] | 20 [19-20] | 18 [18-19] | 18 [17-18] | 17 [16-17] | 17 [16-17] | 16 [16-17] |
|  | **<60 bpm** | **CNR** | 16 [16-17] | 21 [19-23] | 20 [18-22] | 19 [17-20] | 17 [16-18] | 15 [14-16] | 14 [13-14] | 12 [12-13] |
|  |  | **Attenuation** | 255 [251-259] | 434 [395-472] | 387 [361-413] | 334 [315-353] | 291 [280-302] | 255 [250-261] | 224 [220-228] | 200 [196-204] |
|  |  | **Noise** | 15 [15-16] | 20 [19-22] | 19 [18-20] | 18 [17-19] | 17 [16-18] | 17 [16-17] | 16 [16-17] | 16 [16-17] |
|  | **60-75 bpm** | **CNR** | 15 [13-17] | 19 [17-22] | 18 [15-21] | 16 [14-18] | 15 [13-17] | 14 [12-15] | 12 [11-14] | 11 [11-12] |
|  |  | **Attenuation** | 233 [202-264] | 413 [367-459] | 348 [304-392] | 294 [258-330] | 255 [224-287] | 226 [205-248] | 203 [186-220] | 182 [170-194] |
|  |  | **Noise** | 15 [15-16] | 20 [20-21] | 19 [18-19] | 17 [17-18] | 17 [16-17] | 16 [16-17] | 16 [16-16] | 16 [15-16] |
|  | **>75 bpm** | **CNR** | 11 [10-12] | 16 [14-19] | 15 [13-17] | 14 [12-16] | 12 [11-14] | 11 [10-12] | 10 [9-11] | 9 [8-10] |
|  |  | **Attenuation** | 144 [133-155] | 278 [247-309] | 236 [217-254] | 207 [181-233] | 177 [156-197] | 157 [144-171] | 139 [128-149] | 124 [116-131] |
|  |  | **Noise** | 13 [12-14] | 18 [16-19] | 16 [15-17] | 15 [14-16] | 14 [14-15] | 14 [14-15] | 14 [13-14] | 14 [13-14] |
| **Large** | **0 bpm** | **CNR** | 15 [15-15] | 23 [21-24] | 21 [19-22] | 18 [17-19] | 16 [15-17] | 14 [14-15] | 12 [12-13] | 11 [11-11] |
|  |  | **Attenuation** | 318 [291-345] | 778 [679-876] | 623 [545-700] | 506 [447-566] | 419 [376-462] | 348 [314-383] | 295 [269-321] | 253 [233-274] |
|  |  | **Noise** | 19 [19-20] | 27 [27-28] | 25 [24-25] | 23 [23-23] | 22 [22-22] | 21 [21-22] | 21 [20-21] | 20 [20-21] |
|  | **<60 bpm** | **CNR** | 14 [13-15] | 21 [19-22] | 19 [18-20] | 17 [16-18] | 15 [14-16] | 13 [13-14] | 12 [11-12] | 11 [10-11] |
|  |  | **Attenuation** | 273 [261-285] | 544 [520-568] | 452 [432-472] | 380 [362-398] | 325 [310-339] | 283 [272-293] | 247 [238-255] | 220 [214-225] |
|  |  | **Noise** | 19 [19-20] | 26 [26-27] | 24 [23-25] | 22 [22-23] | 21 [21-22] | 21 [20-21] | 21 [20-21] | 20 [20-21] |
|  | **60-75 bpm** | **CNR** | 11 [10-12] | 16 [14-19] | 15 [13-17] | 14 [12-15] | 12 [11-14] | 11 [10-12] | 10 [9-11] | 9 [8-10] |
|  |  | **Attenuation** | 229 [209-249] | 474 [427-521] | 386 [345-427] | 328 [300-356] | 282 [260-305] | 243 [225-261] | 216 [202-230] | 194 [184-204] |
|  |  | **Noise** | 20 [19-20] | 27 [26-29] | 25 [24-26] | 23 [22-24] | 22 [21-23] | 21 [20-22] | 21 [20-22] | 21 [20-22] |
|  | **>75 bpm** | **CNR** | 8 [7-9] | 12 [11-13] | 11 [10-12] | 10 [9-10] | 9 [8-9] | 8 [8-9] | 7 [7-8] | 7 [7-7] |
|  |  | **Attenuation** | 134 [126-142] | 276 [254-298] | 230 [214-246] | 195 [183-206] | 170 [161-179] | 151 [143-158] | 135 [130-140] | 124 [121-128] |
|  |  | **Noise** | 16 [16-16] | 22 [21-23] | 20 [20-21] | 19 [19-20] | 18 [18-19] | 18 [18-19] | 18 [17-18] | 18 [17-18] |

Supplementary table 1: Mean and standard deviation of CNR, attenuation, and noise at 100% CM dose at 120 kVp.

The conventional reconstructions for each heart rate category and patient size at 120 kVp and 100% CM dose was used as a reference. Abbreviations: bpm: beats per minute, CM: contrast medium, CNR: contrast-to-noise ratio, Conv: conventional, keV: kiloelectron volt, kVp: kilovolt peak.

|  | | | **Full** | | | | | | | |
| --- | --- | --- | --- | --- | --- | --- | --- | --- | --- | --- |
|  |  |  | **140 kVp** | | | | | | | |
|  |  |  | **Conv** | **40 keV** | **45 keV** | **50 keV** | **55 keV** | **60 keV** | **65 keV** | **70 keV** |
| **Small** | **0 bpm** | **CNR** | 25 [24-26] | 41 [39-42] | 36 [34-37] | 32 [31-32] | 27 [27-28] | 24 [23-24] | 21 [21-21] | 19 [18-19] |
|  |  | **Attenuation** | 347 [340-355] | 962 [930-995] | 768 [741-795] | 623 [603-642] | 510 [495-525] | 423 [412-434] | 356 [347-365] | 305 [297-313] |
|  |  | **Noise** | 10 [9-10] | 14 [13-14] | 12 [12-13] | 11 [11-12] | 11 [11-11] | 10 [10-11] | 10 [10-10] | 10 [9-10] |
|  | **<60 bpm** | **CNR** | 21 [19-24] | 34 [31-36] | 30 [28-33] | 27 [25-29] | 24 [22-26] | 21 [19-23] | 19 [17-21] | 17 [15-19] |
|  |  | **Attenuation** | 219 [191-246] | 462 [407-518] | 383 [336-431] | 321 [282-361] | 275 [242-308] | 236 [209-263] | 206 [183-230] | 180 [161-200] |
|  |  | **Noise** | 10 [10-10] | 13 [12-14] | 12 [11-13] | 12 [11-12] | 11 [10-12] | 11 [10-11] | 11 [10-11] | 10 [10-11] |
|  | **60-75 bpm** | **CNR** | 18 [15-20] | 27 [24-30] | 24 [22-26] | 22 [20-24] | 20 [18-21] | 18 [16-19] | 16 [15-18] | 15 [14-16] |
|  |  | **Attenuation** | 181 [155-208] | 366 [332-401] | 302 [274-330] | 253 [227-278] | 220 [200-239] | 189 [172-206] | 169 [151-186] | 150 [135-165] |
|  |  | **Noise** | 10 [10-10] | 13 [13-13] | 12 [12-12] | 11 [11-12] | 11 [10-11] | 10 [10-11] | 10 [10-11] | 10 [10-10] |
|  | **>75 bpm** | **CNR** | 12 [11-12] | 18 [18-19] | 17 [16-17] | 15 [15-16] | 14 [13-15] | 12 [12-13] | 11 [11-12] | 10 [10-10] |
|  |  | **Attenuation** | 123 [115-132] | 257 [251-263] | 219 [213-225] | 189 [181-197] | 165 [154-176] | 143 [135-152] | 127 [119-134] | 113 [106-120] |
|  |  | **Noise** | 10 [10-11] | 14 [13-15] | 13 [12-14] | 12 [12-13] | 12 [11-12] | 12 [11-12] | 11 [11-12] | 11 [11-12] |
| **Medium** | **0 bpm** | **CNR** | 18 [17-18] | 28 [26-30] | 26 [24-28] | 24 [22-25] | 21 [20-22] | 19 [18-19] | 16 [16-17] | 15 [14-15] |
|  |  | **Attenuation** | 326 [320-333] | 926 [894-959] | 741 [714-768] | 599 [577-621] | 490 [471-510] | 407 [390-424] | 342 [327-357] | 292 [278-305] |
|  |  | **Noise** | 14 [13-14] | 18 [18-18] | 16 [16-17] | 15 [15-16] | 15 [15-15] | 14 [14-15] | 14 [14-14] | 14 [14-14] |
|  | **<60 bpm** | **CNR** | 17 [16-18] | 27 [25-29] | 24 [22-25] | 21 [20-23] | 19 [18-20] | 17 [17-18] | 16 [15-17] | 14 [13-15] |
|  |  | **Attenuation** | 227 [220-235] | 472 [445-500] | 392 [373-411] | 331 [314-348] | 283 [268-299] | 247 [236-259] | 219 [206-231] | 194 [186-202] |
|  |  | **Noise** | 13 [13-14] | 18 [17-18] | 16 [16-17] | 16 [15-16] | 15 [15-15] | 14 [14-15] | 14 [14-14] | 14 [14-14] |
|  | **60-75 bpm** | **CNR** | 14 [13-15] | 22 [20-24] | 20 [19-22] | 18 [17-20] | 17 [15-18] | 15 [14-16] | 14 [13-14] | 12 [12-13] |
|  |  | **Attenuation** | 190 [174-206] | 407 [368-447] | 341 [314-367] | 294 [273-316] | 253 [234-271] | 221 [204-238] | 195 [181-208] | 173 [161-184] |
|  |  | **Noise** | 14 [13-14] | 19 [18-19] | 17 [17-17] | 16 [16-16] | 15 [15-15] | 15 [15-15] | 14 [14-14] | 14 [14-14] |
|  | **>75 bpm** | **CNR** | 10 [8-11] | 16 [14-18] | 14 [12-16] | 13 [11-15] | 12 [10-13] | 11 [9-12] | 10 [8-11] | 9 [8-10] |
|  |  | **Attenuation** | 113 [102-123] | 251 [231-271] | 212 [199-226] | 181 [167-194] | 156 [145-167] | 137 [126-149] | 121 [110-131] | 109 [100-119] |
|  |  | **Noise** | 12 [11-13] | 17 [15-19] | 15 [14-17] | 15 [13-16] | 14 [13-15] | 14 [12-15] | 13 [12-15] | 13 [12-14] |
| **Large** | **0 bpm** | **CNR** | 13 [13-13] | 22 [21-24] | 20 [19-22] | 18 [17-19] | 16 [15-17] | 14 [13-15] | 12 [12-13] | 11 [11-11] |
|  |  | **Attenuation** | 269 [253-285] | 779 [702-856] | 628 [568-687] | 510 [465-556] | 421 [385-456] | 352 [324-379] | 298 [276-320] | 262 [240-283] |
|  |  | **Noise** | 18 [18-19] | 27 [26-29] | 24 [23-26] | 23 [21-24] | 21 [20-22] | 20 [20-21] | 20 [19-21] | 20 [19-20] |
|  | **<60 bpm** | **CNR** | 12 [11-12] | 19 [17-22] | 17 [15-19] | 16 [14-17] | 14 [13-15] | 13 [12-13] | 11 [11-12] | 10 [10-10] |
|  |  | **Attenuation** | 226 [218-234] | 497 [424-569] | 411 [359-464] | 351 [315-388] | 304 [280-328] | 267 [253-281] | 234 [224-244] | 208 [203-214] |
|  |  | **Noise** | 19 [19-19] | 26 [25-27] | 24 [23-25] | 23 [22-23] | 22 [21-22] | 21 [21-22] | 21 [20-21] | 20 [20-21] |
|  | **60-75 bpm** | **CNR** | 11 [10-12] | 17 [15-19] | 16 [14-18] | 14 [13-16] | 13 [11-15] | 12 [10-13] | 10 [9-11] | 10 [9-10] |
|  |  | **Attenuation** | 205 [190-221] | 461 [437-486] | 392 [355-428] | 331 [303-359] | 282 [258-307] | 244 [225-262] | 214 [200-228] | 193 [181-205] |
|  |  | **Noise** | 19 [18-20] | 27 [25-28] | 24 [23-26] | 23 [21-24] | 22 [20-23] | 21 [20-22] | 21 [19-22] | 20 [19-22] |
|  | **>75 bpm** | **CNR** | 8 [8-8] | 13 [12-14] | 12 [11-13] | 11 [10-11] | 10 [9-10] | 9 [8-9] | 8 [8-8] | 7 [7-7] |
|  |  | **Attenuation** | 124 [118-130] | 297 [274-321] | 248 [229-268] | 209 [194-225] | 180 [166-195] | 157 [144-169] | 139 [130-149] | 125 [117-132] |
|  |  | **Noise** | 15 [15-16] | 23 [22-24] | 21 [20-22] | 19 [19-20] | 19 [18-19] | 18 [17-19] | 18 [17-18] | 17 [17-18] |

Supplementary table 2: Mean and standard deviation of CNR, attenuation, and noise at 100% CM dose at 140 kVp.

The conventional reconstructions for each heart rate category and patient size at 120 kVp and 100% CM dose was used as a reference. Abbreviations: bpm: beats per minute, CM: contrast medium, CNR: contrast-to-noise ratio, Conv: conventional, keV: kiloelectron volt, kVp: kilovolt peak.

Supplementary table 3: Mean and standard deviation of CNR, attenuation, and noise at 50% CM dose at 120 kVp.

|  | | | **Reduced** | | | | | | | |
| --- | --- | --- | --- | --- | --- | --- | --- | --- | --- | --- |
|  |  |  | **120 kVp** | | | | | | | |
|  |  |  | **Conv** | **40 keV** | **45 keV** | **50 keV** | **55 keV** | **60 keV** | **65 keV** | **70 keV** |
| **Small** | **0 bpm** | **CNR** | 14 [13-14] | 21 [20-23] | 20 [18-21] | 17 [16-18] | 15 [14-16] | 13 [13-14] | 12 [11-12] | 10 [10-11] |
|  |  | **Attenuation** | 192 [173-212] | 449 [403-495] | 361 [325-398] | 294 [265-323] | 243 [220-266] | 203 [184-222] | 173 [157-189] | 149 [135-162] |
|  |  | **Noise** | 11 [11-11] | 13 [12-14] | 13 [12-13] | 12 [12-13] | 12 [11-12] | 12 [11-12] | 12 [11-12] | 12 [11-12] |
|  | **<60 bpm** | **CNR** | 14 [13-14] | 19 [18-21] | 18 [17-18] | 16 [15-17] | 14 [14-15] | 12 [12-13] | 11 [11-12] | 10 [10-11] |
|  |  | **Attenuation** | 154 [148-161] | 275 [251-299] | 236 [221-250] | 203 [192-213] | 173 [166-181] | 149 [143-156] | 133 [128-137] | 118 [114-122] |
|  |  | **Noise** | 11 [11-12] | 14 [14-15] | 13 [13-14] | 13 [12-13] | 12 [12-13] | 12 [12-13] | 12 [11-12] | 12 [11-12] |
|  | **60-75 bpm** | **CNR** | 12 [12-13] | 15 [13-16] | 14 [12-15] | 13 [12-14] | 12 [12-13] | 11 [11-12] | 10 [10-11] | 9 [9-10] |
|  |  | **Attenuation** | 140 [133-148] | 233 [218-248] | 199 [187-212] | 176 [168-184] | 158 [153-162] | 139 [132-145] | 125 [120-130] | 112 [109-115] |
|  |  | **Noise** | 11 [11-12] | 15 [14-16] | 14 [13-14] | 13 [13-14] | 13 [12-13] | 12 [12-13] | 12 [12-12] | 12 [11-12] |
|  | **>75 bpm** | **CNR** | 9 [8-10] | 13 [10-15] | 11 [9-13] | 10 [9-12] | 9 [8-11] | 9 [8-10] | 8 [7-9] | 8 [7-8] |
|  |  | **Attenuation** | 120 [109-131] | 239 [204-274] | 198 [171-226] | 168 [147-190] | 147 [131-163] | 134 [122-145] | 121 [112-130] | 108 [100-115] |
|  |  | **Noise** | 13 [12-15] | 17 [16-19] | 16 [15-18] | 15 [14-17] | 15 [13-16] | 15 [13-16] | 14 [13-16] | 14 [13-15] |
| **Medium** | **0 bpm** | **CNR** | 11 [10-11] | 17 [16-18] | 15 [15-16] | 13 [13-14] | 12 [12-12] | 11 [10-11] | 9 [9-10] | 9 [8-9] |
|  |  | **Attenuation** | 201 [196-205] | 474 [458-490] | 384 [372-395] | 314 [306-322] | 261 [255-267] | 220 [215-226] | 189 [183-194] | 163 [157-170] |
|  |  | **Noise** | 15 [15-15] | 19 [18-20] | 18 [17-19] | 17 [17-18] | 17 [16-17] | 16 [16-16] | 16 [16-16] | 16 [15-16] |
|  | **<60 bpm** | **CNR** | 10 [9-11] | 15 [14-17] | 14 [13-15] | 13 [12-13] | 11 [11-12] | 10 [10-11] | 9 [9-9] | 8 [8-9] |
|  |  | **Attenuation** | 152 [144-160] | 314 [297-331] | 264 [249-278] | 222 [212-232] | 191 [185-197] | 167 [161-173] | 146 [141-151] | 130 [126-135] |
|  |  | **Noise** | 15 [15-16] | 20 [19-21] | 18 [18-19] | 17 [17-18] | 17 [16-17] | 16 [16-17] | 16 [15-17] | 16 [15-16] |
|  | **60-75 bpm** | **CNR** | 9 [9-10] | 13 [11-14] | 12 [11-13] | 11 [10-12] | 10 [9-11] | 9 [9-10] | 8 [8-9] | 8 [7-8] |
|  |  | **Attenuation** | 146 [137-155] | 256 [233-280] | 222 [203-240] | 193 [176-210] | 175 [163-188] | 157 [147-167] | 140 [130-149] | 125 [117-132] |
|  |  | **Noise** | 15 [15-16] | 20 [19-20] | 18 [18-19] | 17 [17-18] | 17 [16-18] | 17 [16-17] | 16 [16-17] | 16 [16-17] |
|  | **>75 bpm** | **CNR** | 9 [8-9] | 13 [11-14] | 12 [11-13] | 11 [10-12] | 10 [8-11] | 9 [8-10] | 8 [7-9] | 7 [6-8] |
|  |  | **Attenuation** | 140 [126-155] | 289 [280-299] | 245 [231-260] | 211 [200-222] | 179 [169-189] | 156 [144-168] | 137 [125-149] | 123 [113-133] |
|  |  | **Noise** | 17 [15-19] | 23 [21-25] | 21 [19-23] | 20 [18-22] | 19 [17-21] | 19 [17-21] | 18 [16-20] | 18 [16-20] |
| **Large** | **0 bpm** | **CNR** | 9 [8-9] | 13 [13-14] | 12 [12-13] | 11 [11-11] | 10 [10-11] | 9 [9-9] | 8 [8-8] | 7 [7-8] |
|  |  | **Attenuation** | 193 [188-200] | 489 [467-511] | 395 [378-412] | 327 [310-343] | 270 [256-284] | 225 [215-235] | 192 [183-202] | 166 [157-175] |
|  |  | **Noise** | 18 [18-19] | 26 [25-27] | 25 [24-26] | 23 [22-24] | 21 [21-22] | 21 [21-22] | 21 [20-21] | 20 [20-21] |
|  | **<60 bpm** | **CNR** | 9 [8-9] | 13 [12-14] | 12 [11-13] | 11 [10-12] | 10 [9-11] | 9 [8-9] | 8 [8-8] | 7 [7-8] |
|  |  | **Attenuation** | 161 [154-168] | 348 [318-378] | 286 [265-308] | 239 [223-255] | 205 [191-218] | 178 [169-186] | 157 [151-163] | 140 [135-146] |
|  |  | **Noise** | 18 [18-19] | 26 [25-28] | 24 [23-25] | 22 [21-23] | 21 [20-22] | 20 [20-21] | 20 [19-20] | 19 [19-20] |
|  | **60-75 bpm** | **CNR** | 8 [8-9] | 13 [11-14] | 12 [11-13] | 10 [10-11] | 9 [9-10] | 8 [8-9] | 8 [7-8] | 7 [7-7] |
|  |  | **Attenuation** | 147 [136-159] | 316 [287-344] | 263 [241-285] | 224 [207-242] | 191 [178-204] | 167 [157-177] | 147 [140-155] | 133 [127-140] |
|  |  | **Noise** | 18 [18-19] | 25 [25-26] | 23 [23-23] | 21 [21-22] | 21 [20-21] | 20 [20-20] | 20 [19-20] | 19 [19-20] |
|  | **>75 bpm** | **CNR** | 6 [6-7] | 9 [8-10] | 9 [8-9] | 8 [7-8] | 7 [6-7] | 6 [6-7] | 6 [6-6] | 5 [5-5] |
|  |  | **Attenuation** | 136 [127-145] | 280 [254-306] | 239 [215-264] | 199 [180-217] | 172 [156-188] | 153 [140-165] | 136 [126-145] | 123 [115-130] |
|  |  | **Noise** | 21 [20-22] | 29 [26-31] | 27 [25-29] | 25 [23-27] | 24 [22-26] | 24 [22-25] | 23 [22-25] | 23 [21-25] |

The conventional reconstructions for each heart rate category and patient size at 120 kVp and 100% CM dose was used as a reference. Abbreviations: bpm: beats per minute, CM: contrast medium, CNR: contrast-to-noise ratio, Conv: conventional, keV: kiloelectron volt, kVp: kilovolt peak.

Supplementary table 4: Mean and standard deviation of CNR, attenuation, and noise at 50% CM dose at 140 kVp.

|  | | | **Reduced** | | | | | | | |
| --- | --- | --- | --- | --- | --- | --- | --- | --- | --- | --- |
|  |  |  | **140 kVp** | | | | | | | |
|  |  |  | **Conv** | **40 keV** | **45 keV** | **50 keV** | **55 keV** | **60 keV** | **65 keV** | **70 keV** |
| **Small** | **0 bpm** | **CNR** | 15 [14-15] | 26 [24-28] | 23 [22-25] | 21 [19-22] | 18 [17-19] | 16 [15-16] | 14 [13-14] | 12 [12-13] |
|  |  | **Attenuation** | 155 [130-179] | 425 [362-489] | 355 [301-408] | 289 [245-332] | 238 [201-275] | 199 [168-230] | 163 [138-188] | 141 [120-162] |
|  |  | **Noise** | 9 [9-10] | 12 [11-13] | 11 [11-11] | 10 [10-11] | 10 [10-10] | 10 [9-10] | 9 [9-10] | 9 [9-10] |
|  | **<60 bpm** | **CNR** | 13 [12-13] | 21 [18-23] | 19 [17-21] | 17 [16-18] | 16 [15-16] | 14 [13-14] | 12 [12-13] | 11 [11-12] |
|  |  | **Attenuation** | 128 [121-134] | 277 [247-308] | 231 [209-254] | 197 [182-212] | 167 [155-179] | 144 [135-152] | 126 [119-133] | 111 [105-117] |
|  |  | **Noise** | 10 [9-10] | 13 [12-13] | 12 [11-12] | 11 [11-11] | 10 [10-11] | 10 [10-10] | 10 [10-10] | 10 [9-10] |
|  | **60-75 bpm** | **CNR** | 12 [11-13] | 19 [16-21] | 17 [13-21] | 16 [12-19] | 14 [13-15] | 13 [10-16] | 11 [9-14] | 10 [9-12] |
|  |  | **Attenuation** | 118 [109-128] | 243 [212-273] | 229 [183-275] | 194 [155-234] | 151 [138-163] | 145 [121-169] | 125 [106-145] | 112 [96-127] |
|  |  | **Noise** | 10 [9-10] | 13 [12-14] | 18 [5-32] | 17 [5-29] | 11 [10-11] | 14 [6-22] | 13 [7-20] | 13 [7-18] |
|  | **>75 bpm** | **CNR** | 8 [8-9] | 13 [12-15] | 12 [11-13] | 11 [10-12] | 10 [9-11] | 9 [8-9] | 8 [8-9] | 7 [7-8] |
|  |  | **Attenuation** | 125 [114-135] | 263 [229-297] | 221 [200-241] | 186 [169-204] | 162 [145-180] | 140 [126-154] | 125 [112-137] | 111 [100-123] |
|  |  | **Noise** | 14 [13-16] | 19 [15-24] | 18 [15-20] | 17 [14-19] | 16 [14-19] | 15 [14-17] | 15 [13-17] | 15 [13-17] |
| **Medium** | **0 bpm** | **CNR** | 10 [10-11] | 19 [16-21] | 17 [15-18] | 15 [13-16] | 13 [12-14] | 12 [11-13] | 10 [10-11] | 9 [9-10] |
|  |  | **Attenuation** | 173 [165-180] | 473 [451-496] | 383 [367-400] | 315 [302-327] | 262 [252-272] | 221 [212-230] | 190 [181-198] | 165 [156-173] |
|  |  | **Noise** | 13 [13-14] | 18 [17-19] | 17 [16-17] | 16 [15-16] | 15 [15-16] | 15 [14-15] | 14 [14-15] | 14 [14-15] |
|  | **<60 bpm** | **CNR** | 10 [10-11] | 18 [17-19] | 16 [16-17] | 15 [14-15] | 13 [13-14] | 12 [11-12] | 11 [10-11] | 9 [9-10] |
|  |  | **Attenuation** | 138 [134-142] | 328 [314-343] | 273 [263-283] | 228 [222-234] | 197 [193-202] | 171 [165-177] | 150 [145-155] | 131 [126-136] |
|  |  | **Noise** | 13 [13-14] | 18 [17-19] | 17 [16-17] | 16 [15-16] | 15 [15-15] | 15 [14-15] | 14 [14-15] | 14 [14-14] |
|  | **60-75 bpm** | **CNR** | 9 [9-10] | 14 [12-16] | 13 [11-15] | 12 [10-13] | 11 [9-12] | 10 [9-11] | 9 [9-10] | 8 [8-9] |
|  |  | **Attenuation** | 123 [117-128] | 275 [244-306] | 227 [203-250] | 191 [174-208] | 166 [151-180] | 148 [138-157] | 131 [126-136] | 118 [115-122] |
|  |  | **Noise** | 13 [13-14] | 19 [18-20] | 17 [16-18] | 16 [16-17] | 16 [15-16] | 15 [15-15] | 15 [14-15] | 14 [14-15] |
|  | **>75 bpm** | **CNR** | 7 [7-8] | 12 [11-13] | 11 [10-12] | 10 [9-11] | 9 [8-10] | 8 [7-9] | 7 [7-8] | 7 [6-7] |
|  |  | **Attenuation** | 124 [116-132] | 278 [248-308] | 238 [216-261] | 201 [185-216] | 173 [161-185] | 150 [140-160] | 133 [125-141] | 120 [113-127] |
|  |  | **Noise** | 17 [16-19] | 23 [21-25] | 21 [20-23] | 20 [19-22] | 19 [18-21] | 19 [17-21] | 19 [17-20] | 18 [17-20] |
| **Large** | **0 bpm** | **CNR** | 8 [7-8] | 13 [11-14] | 12 [11-13] | 11 [10-12] | 10 [9-10] | 9 [8-9] | 8 [7-8] | 7 [7-8] |
|  |  | **Attenuation** | 161 [148-173] | 454 [426-481] | 367 [344-391] | 301 [281-321] | 250 [232-268] | 211 [195-228] | 181 [166-196] | 159 [147-172] |
|  |  | **Noise** | 19 [18-20] | 29 [28-30] | 26 [25-27] | 24 [23-25] | 23 [21-24] | 22 [21-23] | 21 [20-22] | 21 [20-21] |
|  | **<60 bpm** | **CNR** | 8 [7-8] | 12 [11-14] | 11 [10-13] | 10 [10-11] | 9 [9-10] | 9 [8-9] | 8 [7-8] | 7 [7-7] |
|  |  | **Attenuation** | 135 [133-138] | 307 [280-333] | 257 [239-275] | 223 [211-235] | 192 [185-199] | 168 [165-172] | 149 [145-153] | 133 [130-136] |
|  |  | **Noise** | 18 [17-18] | 26 [24-28] | 23 [22-25] | 22 [21-23] | 21 [20-21] | 20 [19-21] | 19 [19-20] | 19 [19-20] |
|  | **60-75 bpm** | **CNR** | 7 [7-7] | 12 [11-12] | 11 [10-11] | 9 [9-10] | 9 [8-9] | 8 [7-8] | 7 [7-7] | 7 [6-7] |
|  |  | **Attenuation** | 132 [123-140] | 299 [268-330] | 251 [228-275] | 214 [195-233] | 185 [169-201] | 164 [151-176] | 147 [137-156] | 132 [124-141] |
|  |  | **Noise** | 18 [18-18] | 25 [24-26] | 23 [22-24] | 22 [21-22] | 21 [20-21] | 20 [20-21] | 20 [19-20] | 20 [19-20] |
|  | **>75 bpm** | **CNR** | 6 [6-7] | 10 [8-12] | 9 [7-11] | 8 [7-9] | 8 [7-8] | 7 [6-8] | 6 [6-7] | 6 [5-6] |
|  |  | **Attenuation** | 124 [118-130] | 274 [236-312] | 230 [199-261] | 198 [173-222] | 175 [164-186] | 158 [148-167] | 142 [134-150] | 130 [123-138] |
|  |  | **Noise** | 19 [18-20] | 27 [24-29] | 25 [23-27] | 23 [22-25] | 22 [21-24] | 22 [20-24] | 21 [20-23] | 21 [20-23] |

The conventional reconstructions for each heart rate category and patient size at 120 kVp and 100% CM dose was used as a reference. Abbreviations: bpm: beats per minute, CM: contrast medium, CNR: contrast-to-noise ratio, Conv: conventional, keV: kiloelectron volt, kVp: kilovolt peak.
